# Enhancement of Pediocin Production in *Pediococcus pentosaceus* SNSS18 by Exogenous Amino Acids and Nucleobases: A Mechanistic Elucidation

**DOI:** 10.3390/foods15152739

**Published:** 2026-08-04

**Authors:** Xiaojing Guo, Mingzhu Wei, Yifan Mao, Xuguang Qiao, Yiteng Qiao

**Affiliations:** Key Laboratory of Food Nutrition and Health in Universities of Shandong, College of Food Science and Engineering, Shandong Agricultural University, 61 Daizong Street, Tai’an 271018, China; guoxj0610@163.com (X.G.); 13386385347@163.com (M.W.); maoyifana@163.com (Y.M.); xgqiao@sdau.edu.cn (X.Q.)

**Keywords:** *Pediococcus pentosaceus*, pediocin, whole-genome analysis, quorum sensing, amino acids, nucleobases

## Abstract

Amino acids and nucleobases, as essential nutrients for lactic acid bacteria (LAB), play a pivotal role in their growth and metabolism. However, their specific effects on pediocin production and the underlying regulatory mechanisms have not yet been elucidated. This study aimed to identify exogenous amino acids and nucleobases that enhance pediocin production by *Pediococcus pentosaceus* SNSS18, and further analyze their induction mechanisms. Alanine, cysteine, aspartate, methionine, adenine, and uracil significantly promoted pediocin production (*p* < 0.05), achieving a maximum increase of 60.75% in antibacterial activity compared with the control. Genomic analysis revealed the presence of the pediocin PA-1 biosynthesis operon (*pedABCD*) and the LuxS/AI-2 quorum-sensing (QS) system in *P. pentosaceus* SNSS18. Notably, methionine (0.5 g/L), cysteine (0.5 g/L), aspartate (1 g/L), and uracil (1.5 g/L) enhanced AI-2 signal activity and upregulated the expression of key QS genes (*luxS* and *pfs*), suggesting that their promotive effects are likely linked to the LuxS/AI-2 QS system. Exogenous supplementation with AI-2 further verified that the LuxS/AI-2 QS system is a key pathway regulating pediocin production in *P. pentosaceus* SNSS18. However, the regulatory mechanisms underlying the promotive effects of alanine and adenine remain to be elucidated. These findings provide new insights into the regulation of pediocin biosynthesis and highlight the potential of exogenous amino acid and nucleobase supplementation to markedly enhance pediocin production for food biopreservation, while supporting the development of *P. pentosaceus* SNSS18 as a probiotic strain.

## 1. Introduction

In recent years, growing public concern over intestinal health and the frequent occurrence of foodborne diseases have heightened interest in lactic acid bacteria (LAB) and their bioactive secondary metabolites. These metabolites, while not essential for growth, enhance competitive fitness and benefit both hosts and food systems [1,2]. Bacteriocins, ribosomally synthesized antimicrobial peptides by LAB, are readily degraded by gastrointestinal proteases and exhibit a low risk of resistance development, making them attractive natural food preservatives [3,4]. Pediocin, a class IIa bacteriocin mainly produced by *Pediococcus* spp., can inhibit a broad spectrum of foodborne pathogens including *Listeria monocytogenes* and *Enterococcus faecalis*, with particularly high potency against *L. monocytogenes* even at nanomolar concentrations [5,6,7,8]. However, its industrial application is limited by low natural yield and poorly understood biosynthetic regulatory mechanisms.

LAB growth and bacteriocin synthesis are tightly regulated by quorum sensing (QS) systems, which are generally categorized into intraspecies-specific and interspecies-general types [9]. The former relies on species-specific autoinducing peptides (AIPs) and regulates collective behaviors within the same species, such as biofilm formation and bacteriocin production. The latter centers on the LuxS/autoinducer-2 (AI-2) system, which facilitates cross-species communication among different bacteria and even between bacteria and fungi through the secretion of the universal signaling molecule AI-2 [10,11]. This system serves as an important regulatory network for LAB to adapt to complex microecological environments, such as fermented food systems and the human intestinal tract [12]. AI-2 synthesis is catalyzed by the enzyme LuxS within the activated methyl cycle (AMC) and is closely coupled to the metabolism of amino acids (especially sulfur-containing types) and nucleotides [13].

Various strategies have been developed to enhance bacteriocin production, including optimization of culture conditions and medium composition, metabolic regulation, QS regulation, and genetic engineering [14,15]. Among these strategies, exogenous supplementation with amino acids and nucleobases has attracted increasing attention as a simple, food-grade, and cost-effective approach that can be readily implemented in industrial fermentation processes. As essential small molecules in microorganisms, amino acids and nucleobases function as macromolecular building blocks and key metabolic regulators, and their exogenous supplementation has been demonstrated to enhance LAB growth and bacteriocin production [16,17]. For example, the addition of guanine and tyrosine increased the production of pediocin by *Pediococcus acidilactici* [18], while glutamine, glutamate, and 5-methylcytosine were found to enhance Plantaricin Q7 production by *Lactiplantibacillus plantarum* [19]. Earlier studies also demonstrated that amino acid supplementation stimulated nisin and pediocin production and suggested that this effect was more likely associated with an auto-stimulatory mechanism than with a purely nutritional phenomenon [20]. Although these findings highlight the potential of amino acids and nucleobases as fermentation enhancers, their beneficial effects may involve both nutritional supplementation and additional regulatory mechanisms, such as auto-stimulatory regulation. However, the specific molecular mechanisms underlying bacteriocin enhancement remain largely unresolved. Notably, emerging evidence indicates that these compounds can also exert regulatory effects by influencing QS systems. A 25 mM cysteine was shown to upregulate AI-2 synthesis and enhance the LuxS/AI-2 system in *L. plantarum* SS-128, thereby strengthening its antimicrobial activity [21]. Alanine can function as a QS signal molecule, significantly promoting the growth of *Lactobacillus acidophilus* and enhancing its survival in gastrointestinal environments [22]. Furthermore, nucleotides can bind to the LuxS protein, stimulate AI-2 secretion, and consequently enhance the growth and pathogen-antagonistic activity of *Lacticaseibacillus casei* [23].

Despite these advances, how exogenous amino acids and nucleobases regulate pediocin production is still poorly understood. To address this gap, this study focused on *Pediococcus pentosaceus* SNSS18, a strain harboring the *pedABCD* gene cluster for pediocin PA-1 biosynthesis [24]. Despite its genetic potential for pediocin biosynthesis, SNSS18 exhibits relatively low pediocin production, and the regulatory mechanisms underlying its biosynthesis remain unclear. Here, we investigated the effects of exogenous amino acids and nucleobases on pediocin production in SNSS18 and explored the potential regulatory mechanisms from the QS perspective. This work provides novel insights into the regulatory network controlling pediocin biosynthesis and provides a theoretical basis for the industrial production of pediocin.

## 2. Materials and Methods

### 2.1. Bacterial Strains and Culture Conditions

*P. pentosaceus* SNSS18, a pediocin-producing LAB strain, was isolated from Chinese pickles. *Enterococcus faecalis* SNSS1133 was sourced from the College of Food Science and Engineering, Shandong Agricultural University. The culture medium of de Man, Rogosa, and Sharpe (MRS) was purchased from Hopebio (Qingdao, China) and sterilized at 121 °C for 20 min. *P. pentosaceus* SNSS18 and *E. faecalis* SNSS1133 were inoculated into MRS medium and cultured twice at 37 °C for activation prior to use.

### 2.2. Effect of Amino Acids and Nucleobases on Bacteriocin Production

Based on previously reported amino acids and nucleobases known to enhance bacteriocin production, together with their potential roles in microbial metabolism and QS [19,20,21], nine compounds were selected to investigate their effects on pediocin production by *P. pentosaceus* SNSS18: alanine (Ala), threonine (Thr), aspartate (Asp), cysteine (Cys), methionine (Met), glycine (Gly), adenine (Ade), uracil (Ura), and 5-methylcytosine (5 mC). Stock solutions of each amino acid and nucleobase were prepared in sterile deionized water and sterilized by passage through 0.22 µm membrane filters. Each sterile stock solution was individually supplemented into MRS medium to a final concentration of 0.5 g/L. The medium was then inoculated with 2% (*v*/*v*) of a 16 h seed culture of *P. pentosaceus* SNSS18 containing approximately 5 × 10^8^ CFU/mL and incubated at 37 °C for 24 h for the initial screening of the effects of these compounds on pediocin production. The cell-free supernatants were then collected and tested for antibacterial activity against *E. faecalis* SNSS1133 using the agar well diffusion method. The results were quantified and expressed as arbitrary units per milliliter (AU/mL) [25].

### 2.3. Monitoring of Bacterial Growth

The aforementioned amino acids and nucleobases with inducing effects were added to the culture medium at their optimal concentrations. Then *P. pentosaceus* SNSS18 was inoculated, followed by incubation at 37 °C for 24 h. Subsamples were taken at different time points (0, 2, 6, 8, 10, 12, 16, and 24 h), and their absorbance was measured at 600 nm using an M5 multifunctional microplate reader (Molecular Devices, San Jose, CA, USA) to monitor the growth of the strain.

### 2.4. Whole-Genome Sequencing and Analysis

The bacterial culture of *P. pentosaceus* SNSS18 was centrifuged at 8000× *g* for 15 min at 4 °C to collect the bacterial pellet, which was then snap-frozen in liquid nitrogen. The samples were immediately sent to Majorbio Bio-pharm Technology Co., Ltd. (Shanghai, China) for genomic DNA extraction, PCR amplification, and recovery of the products. The integrity of the DNA was initially assessed by agarose gel electrophoresis, and DNA purity was determined using either the TBS380 or Nanodrop2500 systems. Whole-genome sequencing was performed using a combination of Illumina (second-generation) and PacBio (third-generation) sequencing platforms.

Coding sequences (CDSs) within the genome were predicted using Glimmer v3.02, Prodigal v2.6.3, and GeneMarkS v4.28 software. The CDSs were then subjected to BLAST+ (v2.13.0) searches against the Non-Redundant Protein (NR), Pfam, Clusters of Orthologous Groups of Proteins (COG), Gene Ontology (GO), and Kyoto Encyclopedia of Genes and Genomes (KEGG) databases for functional annotation. Biosynthetic gene clusters (BGCs) for secondary metabolites were predicted using AntiSMASH 7.0.0. Furthermore, gene clusters involved in bacteriocin biosynthesis were analyzed using BAGEL4 software (v4.0). The BioProject accession number for *P. pentosaceus* SNSS18 is PRJNA1390871, and the BioSample accession number is SAMN54208042.

### 2.5. Detection of AI-2 Activity

As described in Section 2.3, *P. pentosaceus* SNSS18 was cultured in MRS medium supplemented with amino acids and nucleobases. Subsamples were taken at 8 h, 16 h, and 24 h and centrifuged at 8000× *g* for 10 min to obtain the supernatant. It was then adjusted to pH 7.0 with 1 M NaOH and sterile-filtered through a 0.22 µm cellulose acetate membrane. AI-2 activity was detected using the reporter strain *Vibrio harveyi* BB170, as described previously [26]. *V. harveyi* BB170 was cultured in AB medium at 30 °C with shaking at 120 rpm until OD_600_ reached 0.9–1.1. The culture was then diluted 1:5000 in fresh sterile AB medium to obtain a suspension of approximately 10^5^ CFU/mL. Test samples, sterile AB medium (negative control), and sterile MRS medium (medium control) were mixed with the diluted *V. harveyi* suspension at a 1:100 ratio and incubated with shaking at 30 °C for 0–6 h. Luminescence was measured every 30 min using an EnSpire microplate reader (PerkinElmer, Waltham, MA, USA) in chemiluminescence mode. AI-2 activity was expressed as relative luminescence units (RLUs).

### 2.6. Gene Expression Analysis by RT-qPCR

Total RNA was extracted using the Total RNA Extraction Kit (New Cell & Molecular, Suzhou, China), and then immediately reverse-transcribed into cDNA using the Evo M-MLV RT Mix Kit with gDNA Clean for qPCR (Accurate Biotechnology, Changsha, China). Primers for the *pedA*, *luxS*, and *pfs* genes were designed based on their respective sequences obtained from whole-genome analysis, with detailed primer sequences provided in Table 1. RT-qPCR was performed using the SYBR Green Pro Taq HS qPCR Kit (Accurate Biotechnology, Changsha, China) with cDNA as the template. The *16S rRNA* gene was used as the internal reference, and the relative gene expression levels were calculated using the 2^−ΔΔCt^ method.

### 2.7. Detection of AI-2 Induction Effect

The precursor of the AI-2 signaling molecule, DPD (4,5-dihydroxy-2,3-pentanedione; Omm Scientific, Dallas, TX, USA), was filter-sterilized through a 0.22 µm cellulose acetate membrane and added to the culture medium at final concentrations of 0, 5, 10, 20, and 40 µM, respectively. Subsequently, *P. pentosaceus* SNSS18 was inoculated and cultured at 37 °C. The antibacterial activity, AI-2 activity, and the relative expression levels of the *pedA*, *luxS*, and *pfs* genes were measured accordingly.

### 2.8. Statistical Analysis

All experiments were performed with three independent biological replicates. Data are expressed as mean ± standard deviation (SD). Statistical analysis was performed using one-way ANOVA followed by Duncan’s test in SPSS 22.0 software, with significance set at *p* < 0.05. Figures were generated using GraphPad Prism 9.5 software.

## 3. Results

### 3.1. Effect of Exogenous Amino Acids and Nucleobase on Pediocin Production

An initial screening was first performed by individually supplementing the nine selected amino acids and nucleobases into MRS medium at a final concentration of 0.5 g/L. As shown in Figure S1, six of these compounds—alanine, cysteine, aspartate, methionine, adenine, and uracil—were found to significantly promote pediocin production by *P. pentosaceus* SNSS18 (*p* < 0.05). Therefore, the effects of different concentrations of these compounds on pediocin production were further investigated. The optimal concentrations were determined as follows: 0.5 g/L for alanine, methionine, and cysteine; 1 g/L for aspartate and adenine; and 1.5 g/L for uracil (Table 2). The maximum antibacterial activity reached 2557.68 AU/mL, representing a 60.75% increase compared to the control group, demonstrating a significant improvement in practical yield.

### 3.2. Growth Characteristics of P. pentosaceus SNSS18 Under the Effect of Amino Acids and Nucleobases

**Figure 1:**
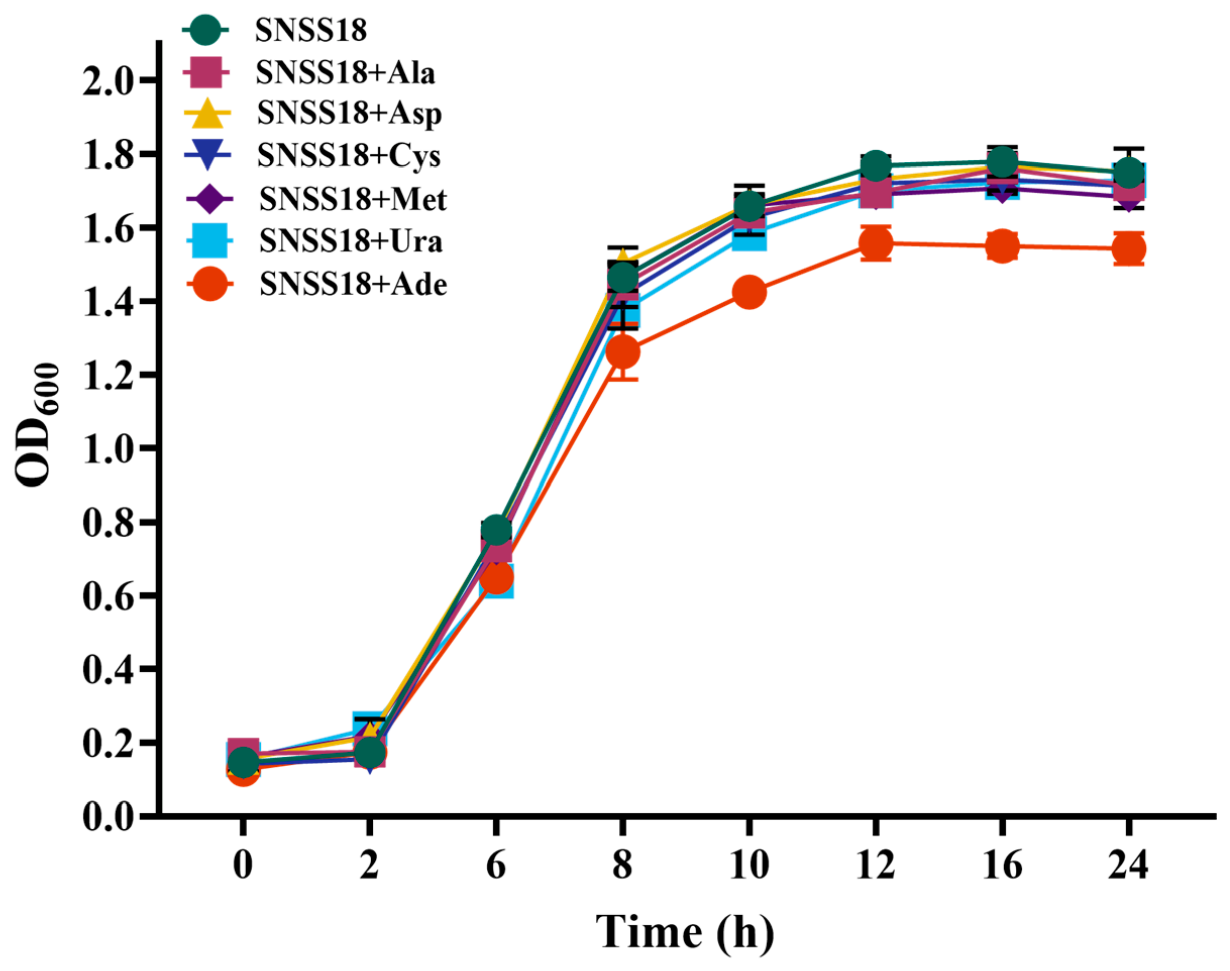
Effects of exogenous amino acids and nucleobases on the growth of *P. pentosaceus* SNSS18.

The growth of *P. pentosaceus* SNSS18 was then examined following separate supplementation with alanine, methionine, and cysteine (each 0.5 g/L), aspartate and adenine (each 1 g/L), and uracil (1.5 g/L) (Figure 1). The growth of *P. pentosaceus* SNSS18 was not significantly affected by the addition of alanine, aspartate, cysteine, or methionine. In contrast, the addition of adenine or uracil significantly inhibited bacterial growth. These findings indicated that the six amino acids and nucleobases did not enhance pediocin production in *P. pentosaceus* SNSS18 by promoting bacterial growth. Although secondary metabolite production in LAB is associated with cell density, it is not entirely dependent on growth conditions [15].

### 3.3. Whole-Genome Analysis of P. pentosaceus SNSS18

#### 3.3.1. Genomic Characteristics of *P. pentosaceus* SNSS18

**Figure 2:**
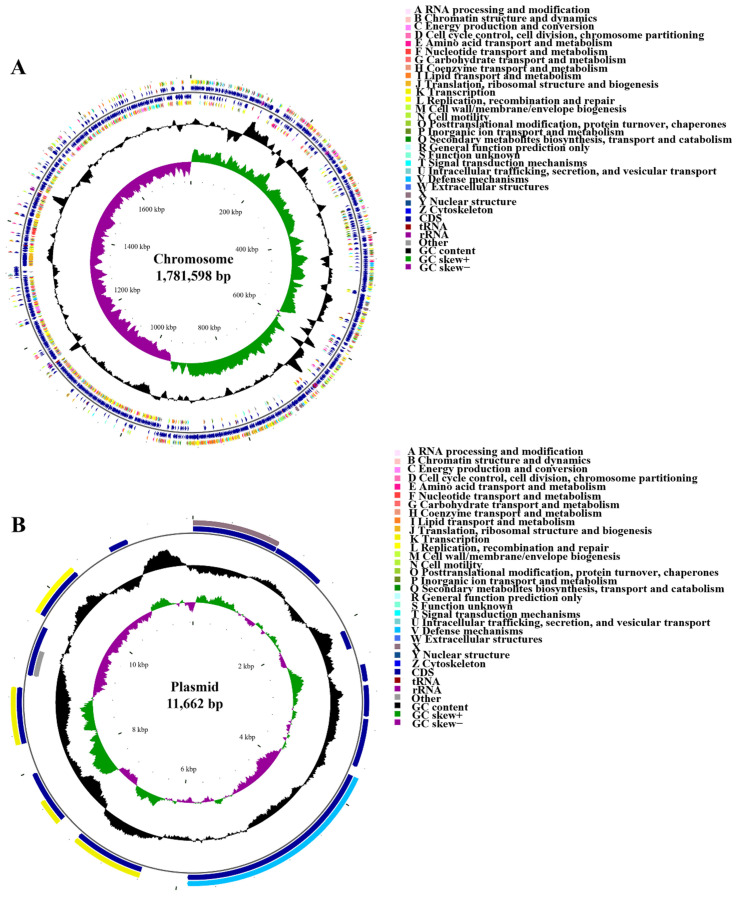
Chromosome (**A**) and plasmid (**B**) circular maps of *P. pentosaceus* SNSS18.

The genomic characteristics of *P. pentosaceus* SNSS18 were investigated using a combined strategy of second-generation and third-generation sequencing. Circular genome maps were shown in Figure 2A,B. The complete genome was found to consist of one circular chromosome (1,781,598 bp) and one circular plasmid (11,662 bp), with GC contents of 37.25% and 35.35%, respectively. Using Glimmer, GeneMarkS, and Prodigal, a total of 1804 CDSs were predicted, with a total length of 1,578,624 bp, accounting for 88.03% of the entire genome. Additionally, 55 tRNA, 15 rRNA, and 27 sRNA genes were identified.

#### 3.3.2. Phylogenetic Tree Analysis of *P. pentosaceus* SNSS18

A phylogenetic tree was constructed using the neighbor-joining method in MEGA 6.0 based on the 16S rRNA gene sequence. As shown in Figure S2A, SNSS18 exhibited the highest sequence identity (99.94%) and 100% query coverage with *Pediococcus pentosaceus* DSM 20336, along with a high identity (98.35%) with *P. acidilactici* DSM 20284. Given the strong conservation of the 16S rRNA gene between these two species [27], phylogenetic analysis based solely on 16S rRNA exhibits limited discriminatory power for closely related strains.

To overcome the limitations of 16S rRNA-based phylogeny, phylogenetic analysis based on housekeeping genes offers higher resolution for differentiating and identifying closely related strains [28,29]. A total of 31 housekeeping genes (including *dnaG*, *frr*, *rpoB*, *rpsB*, and *rpsC*) were identified in the SNSS18 genome, and their concatenated sequences were used to construct a phylogenetic tree. Consistent with the 16S rRNA gene-based analysis, SNSS18 showed the highest sequence similarity (99.7%) with *P. pentosaceus* DSM 20336, markedly higher than with *P. acidilactici* DSM 20284 (91.9%) (Figure S2B). Therefore, strain SNSS18 was identified as *Pediococcus pentosaceus*.

#### 3.3.3. Genomic Functional Annotation of *P. pentosaceus* SNSS18

Functional annotation of the SNSS18 genome was accomplished through multiple databases, including NR, Pfam, COG, GO, and KEGG (Table S1). The highest number of genes was annotated in the NR database, with 1801 genes annotated. Whereas 1545, 1481, 1072, and 1337 genes were annotated in the Pfam, COG, GO, and KEGG databases, respectively.

**Figure 3:**
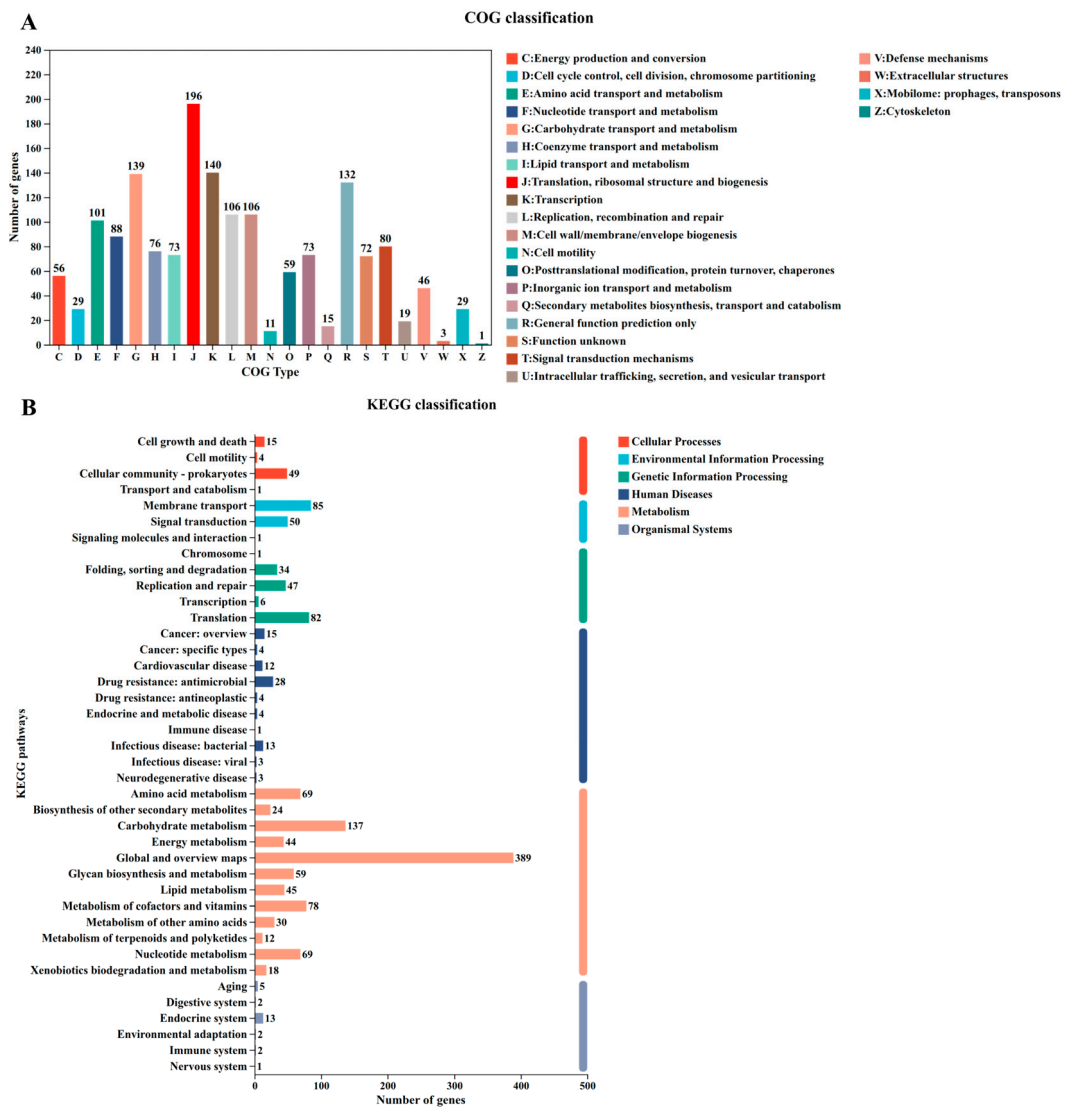
Results of COG (**A**) and KEGG (**B**) annotation of *P. pentosaceus* SNSS18.

COG functional classification identified 1481 genes (82.1%), which were annotated into 23 COG categories (Figure 3A). The largest category was translation, ribosomal structure and biogenesis, comprising 196 genes. This was followed by transcription and carbohydrate transport and metabolism, with 140 and 139 annotated genes, respectively. Additionally, 106 genes were annotated for cell wall/membrane/envelope biogenesis, and 101 genes for amino acid transport and metabolism. Meanwhile, KEGG functional classification identified 1337 genes. Among these, 137 genes were involved in carbohydrate metabolism, 85 genes were related to membrane transport, and 82 genes were associated with translation (Figure 3B). These results indicate that *P. pentosaceus* SNSS18 exhibits active transcription, translation, carbohydrate metabolism, and membrane transport activities, which may contribute to the synthesis of various secondary metabolites.

#### 3.3.4. Genomic Safety Assessment of *P. pentosaceus* SNSS18

The safety of *P. pentosaceus* SNSS18 was evaluated by predicting potential virulence factor genes and antibiotic resistance genes based on its whole-genome sequence. Alignment with the Virulence Factor Database (VFDB) at thresholds of 75% identity and coverage revealed no predicted virulence factors [30]. Meanwhile, antibiotic resistance genes were annotated through homology alignment with the Comprehensive Antibiotic Resistance Database (CARD). The genome of *P. pentosaceus* SNSS18 harbored a single gene conferring resistance to elfamycin antibiotics, with a sequence identity of only 76.1%. This gene was chromosomally encoded, and no antibiotic resistance genes were detected on plasmids. Furthermore, analysis of the flanking genomic region revealed no adjacent mobile genetic elements, such as transposases or integrative elements, associated with this resistance locus. Therefore, the antibiotic resistance of *P. pentosaceus* SNSS18 is likely intrinsic and non-transferable [31]. Previous studies have indicated that many LAB strains exhibit intrinsic resistance to elfamycin antibiotics [32]. Collectively, the genomic analysis supports that *P. pentosaceus* SNSS18 is a safe probiotic strain with promising potential in food industries.

#### 3.3.5. Prediction of Bacteriocin and Quorum Sensing Genes

**Figure 4:**
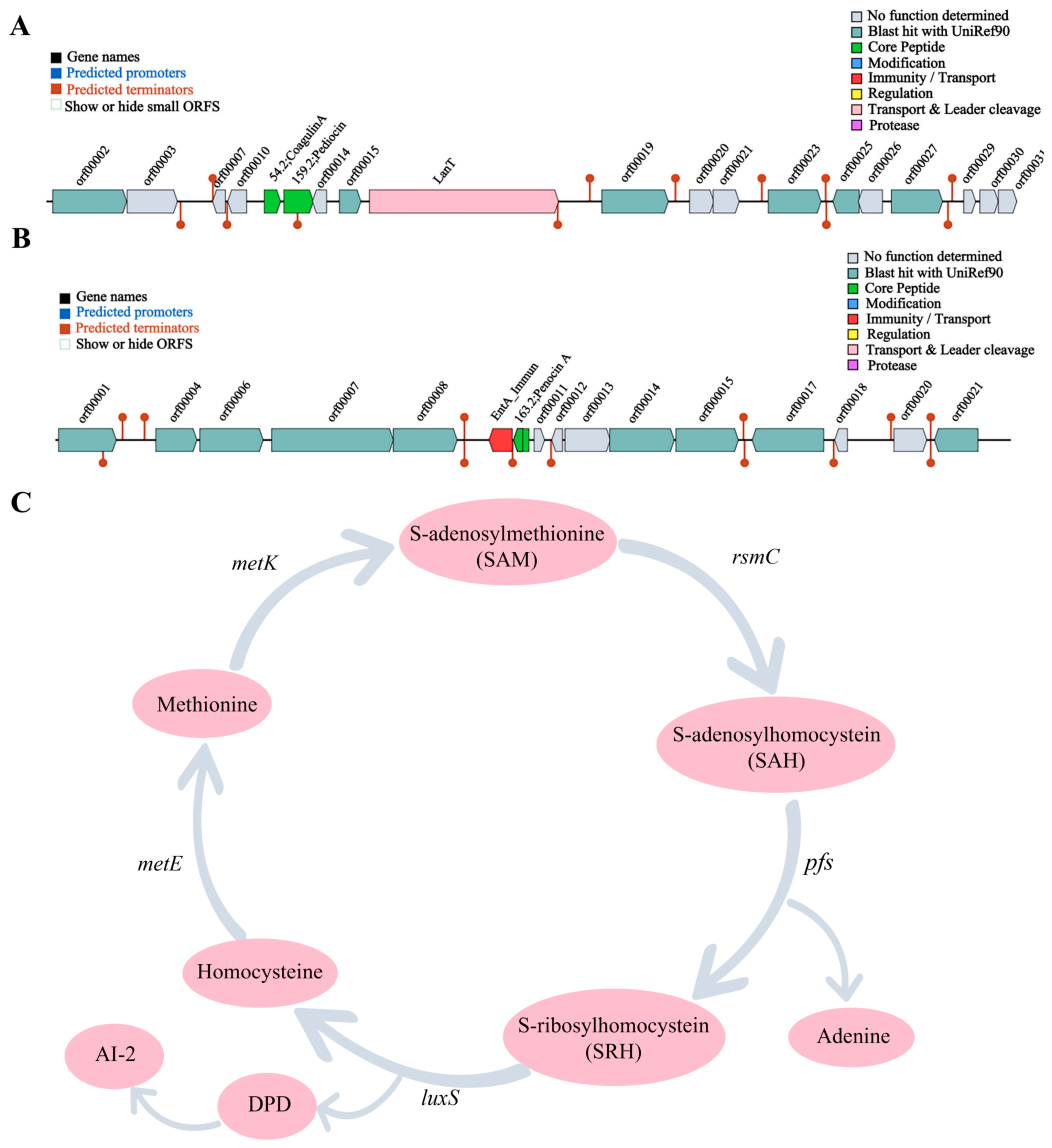
Two bacteriocin synthesis gene operons of *P. pentosaceus* SNSS18 (**A**,**B**); schematic diagram of the LuxS/AI-2 QS system (**C**).

Based on functional annotation, further analysis using antiSMASH and BAGEL4 was conducted to investigate gene clusters involved in the biosynthesis of ribosomally synthesized and post-translationally modified peptides (RiPPs) and bacteriocins. Two operons encoding class II bacteriocins were identified in the *P. pentosaceus* SNSS18 genome. The first operon was located on the plasmid, with pediocin PA-1 and coagulin as the core peptides. Downstream genes encoding the bacteriocin immunity protein PedB, the ABC transporter BlpA (PedD), and its accessory factor PedC constitute a complete pediocin synthesis cluster (Figure 4A). The *pedA* gene encodes a 61-amino-acid precursor peptide featuring a typical double-glycine leader sequence and the class II bacteriocin conserved motif -YGNGV- at its N-terminus. The precursor is recognized and cleaved at the double-glycine site by the ABC transporter PedD, assisted by PedC, and then exported to exert its function [33]. Furthermore, multiple putative transcriptional regulator genes were found downstream of the pediocin synthesis gene. Although no histidine kinase gene was detected, an integral membrane protein gene that may participate in signal transduction was identified. These response regulators, together with the bacteriocin genes, potentially form a self-contained genetic module, allowing the plasmid to function autonomously in a new host without chromosomal regulatory input [34]. The second operon was located on the chromosome, with class II bacteriocins hiracin-JM79 and penocin A as the core peptides. Flanking the bacteriocin gene were genes encoding hiracin-JM79 immunity proteins, histidine protein kinases, LytTR family response regulators, and ABC transporters, all of which contribute to the biosynthesis and regulation of bioactive proteins (Figure 4B). Additionally, antiSMASH analysis identified a putative chromosome-encoded biosynthetic gene cluster similar to the reported fusaricidin B biosynthetic region (Figure S3). Jiang et al. [35] predicted bacteriocin operons across 74 *P. pentosaceus* strains and identified four distinct operon types. Besides the pediocin PA-1 and penocin predicted in SNSS18, operons for enterolysin A and Bac were also reported. The plasmid-encoded *pedABCD* cluster of SNSS18 showed high similarity to previously characterized pediocin PA-1 biosynthetic regions carried by plasmids in *P. acidilactici* PAC1.0 and *P. pentosaceus* NCDC 273 [36], suggesting that plasmid-associated organization of the *pedABCD* cluster is conserved among several pediocin PA-1-producing *Pediococcus* strains.

QS systems regulating bacteriocin synthesis in LAB are classified into intraspecific and interspecific types. In Gram-positive bacteria, intraspecific QS is typically composed of AIP and a two-component regulatory system (TCS). In this study, no genes encoding AIP or TCS were identified upstream or downstream of the pediocin PA-1 gene cluster, which is consistent with previous reports [24,37]. The regulatory mechanism for pediocin PA-1 biosynthesis has not yet been fully elucidated. However, whole-genome analysis identified *luxS* (gene1650, 474 bp) and *pfs*/*mtnN* (gene1180, 702 bp), which encode the key enzymes responsible for synthesizing the interspecies QS signal molecule AI-2. As a universal QS signaling molecule used by both Gram-negative and Gram-positive bacteria, AI-2 plays an important role in regulating bacterial interactions, growth, and metabolism. In the methylation cycle, S-adenosylhomocysteine (SAH) is converted to S-ribosylhomocysteine (SRH) and adenine by Pfs (5′-methylthioadenosine/S-adenosylhomocysteine nucleosidase). SRH is then cleaved by the S-ribosylhomocysteine lyase LuxS into DPD, the precursor of AI-2 [38]. DPD is unstable and can spontaneously cyclize to form AI-2. Further examination of the whole-genome annotation revealed that, besides luxS and pfs, strain SNSS18 also possessed other genes involved in the AI-2 biosynthesis pathway, including *metK* and *rsmC*, all of which were located on the chromosome. The AI-2 synthesis pathway was summarized in Figure 4C.

### 3.4. Effect of Amino Acids and Nucleobases on AI-2 Activity in P. pentosaceus SNSS18

**Figure 5:**
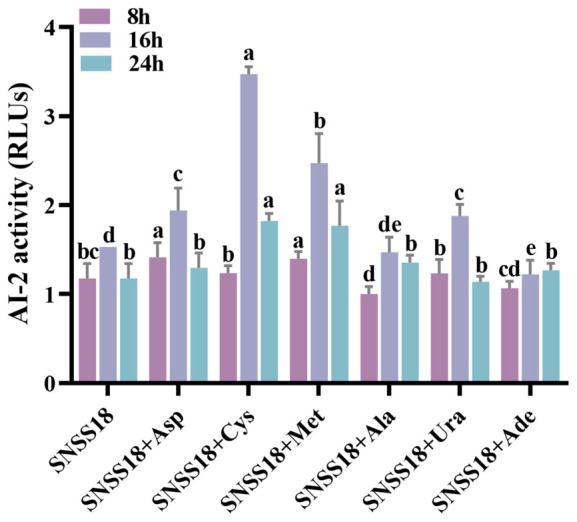
AI-2 activity of *P. pentosaceus* SNSS18 under different exogenous substance treatments. Different letters indicate significant differences among different treatments within the same sampling time point (*p* < 0.05).

The tested exogenous amino acids and nucleobases promoted pediocin production by *P. pentosaceus* SNSS18 without stimulating bacterial growth, suggesting that their effects may involve regulatory mechanisms rather than simple nutrient supplementation. Given that amino acids and nucleobases are closely associated with QS systems, it was investigated whether their induction effects were mediated by the LuxS/AI-2 QS pathway. AI-2 activity was measured following individual supplementation with aspartate, cysteine, methionine, alanine, uracil, or adenine. As shown in Figure 5, AI-2 activity increased over incubation time, peaked at 16 h (late exponential phase), and then declined, which was consistent with previous studies [39]. Among the tested exogenous substances, cysteine and methionine significantly enhanced AI-2 activity, whereas alanine and adenine showed no significant effect and even exhibited slight inhibitory effects at certain time points. Interestingly, aspartate and uracil also significantly stimulated AI-2 activity, though their inductive effects were weaker than those of cysteine and methionine. This may be attributed to the direct involvement of cysteine and methionine in the AMC pathway, which is central to the LuxS/AI-2 QS system [21]. The positive effects of aspartate and uracil are likely mediated through metabolic crosstalk with the AMC pathway and LuxS/AI-2 system. Specifically, aspartate acts as a crucial precursor for methionine, providing carbon skeletons for the AMC pathway and thereby indirectly promoting AI-2 synthesis [40,41]. Previous studies have reported that uracil could enhance the LuxS/AI-2 QS system by promoting the uridine monophosphate (UMP) synthesis pathway [42]. As a core molecule in pyrimidine metabolism, UMP may indirectly regulate the LuxS/AI-2 system through metabolic networks. Further investigation is required to elucidate the metabolic crosstalk between amino acid/pyrimidine metabolism and QS.

In contrast, alanine and adenine significantly enhanced pediocin production without affecting AI-2 activity, pointing to the existence of alternative mechanisms. Previous studies have proposed that alanine may function as a signal molecule to enhance cell–cell communication and participate in AI-2 synthesis [22]. However, in the present study, alanine supplementation did not significantly alter AI-2 activity, indicating that this regulatory mechanism may be strain-dependent or not the predominant pathway in *P. pentosaceus* SNSS18. Because adenine (and similarly uracil) markedly restricted the growth of *P. pentosaceus* SNSS18 while still promoting pediocin production, its stimulatory effect may be partially associated with a physiological stress response triggered by growth inhibition [43]. For alanine, whose effect was growth-neutral and AI-2-independent, alternative metabolic regulatory pathways may be involved. Alanine can be transaminated to pyruvate, potentially affecting glycolytic flux and the intracellular pool of branched-chain amino acids [16]. Changes in branched-chain amino acid availability may modulate the activity of CodY, whereas altered glycolytic intermediates could affect CcpA-mediated carbon catabolite regulation [44]. These global nutrient-sensing regulators, in turn, could influence secondary metabolite production, including bacteriocins [45]. Nevertheless, these possibilities remain speculative and require further study.

### 3.5. Effect of Amino Acids and Nucleobases on Gene Expression of P. pentosaceus SNSS18

**Figure 6:**
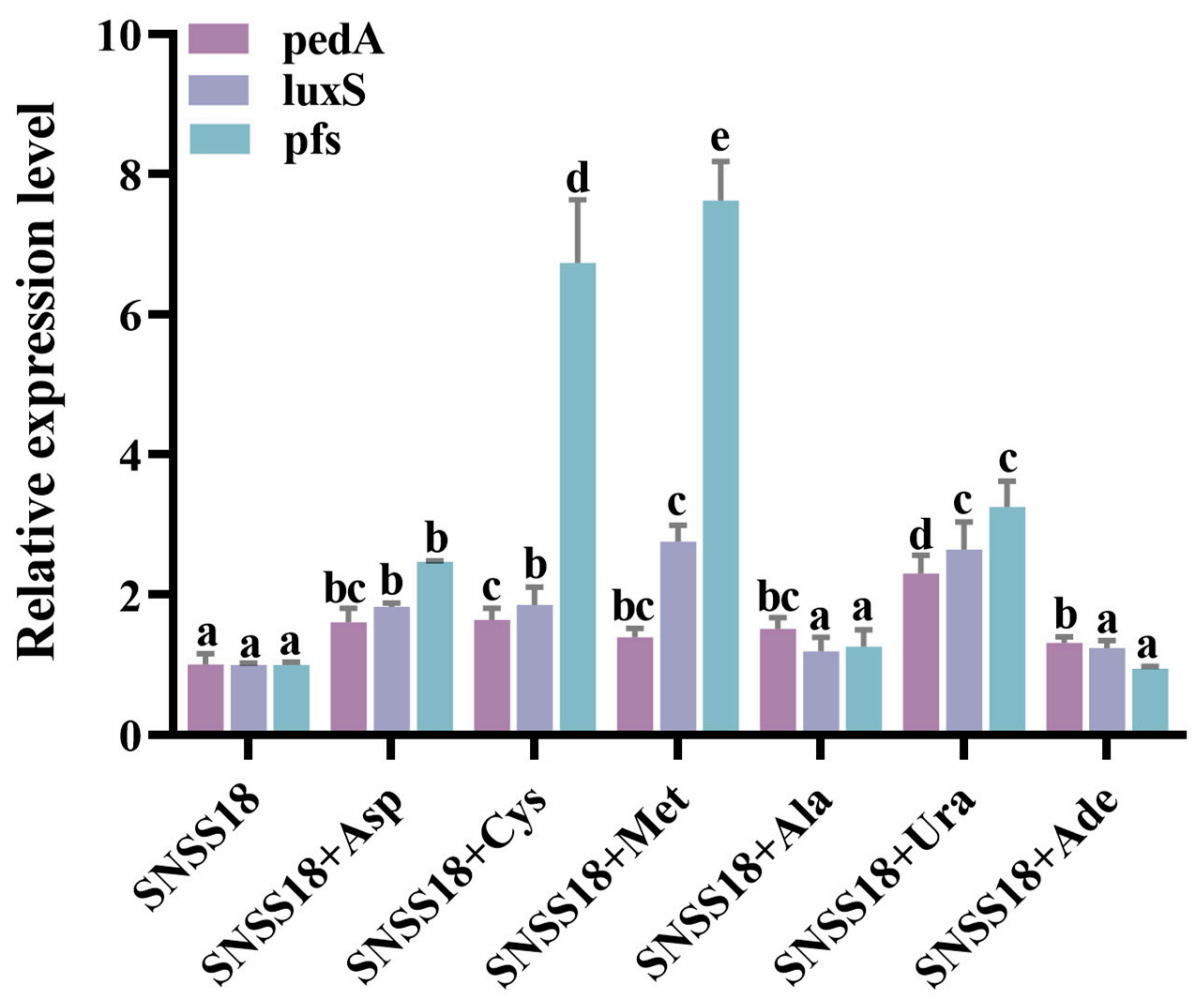
The relative expression of genes under the effect of different exogenous substances. Different letters indicate significant differences among different treatments within the same sampling time point (*p* < 0.05).

The expression of the pediocin gene *pedA* and key genes of the LuxS/AI-2 QS system (*luxS* and *pfs*) was analyzed following treatment with aspartate, cysteine, methionine, alanine, uracil, or adenine (Figure 6). *pedA* expression was significantly upregulated by all six exogenous substances, consistent with the changes in antibacterial activity. Alanine and adenine had no significant effects on the expression of *luxS* and *pfs*. In contrast, cysteine, methionine, aspartate, and uracil significantly enhanced the expression of *luxS* and *pfs*, with methionine and cysteine showing the strongest effects. Notably, the expression of *pfs* was markedly upregulated by methionine and cysteine, with a >6-fold increase in transcript level. These results were in line with the alterations in AI-2 activity, confirming that aspartate, cysteine, methionine, and uracil could activate the LuxS/AI-2 QS system.

Based on these findings, the enhancement of pediocin production by aspartate, cysteine, methionine, and uracil was presumed to be associated with the increase in AI-2 activity. To test this hypothesis, it was next examined whether exogenous addition of the AI-2 precursor DPD could enhance pediocin production, AI-2 activity, and the expression of related genes.

### 3.6. Effect of Exogenous DPD on Bacteriocin Production in P. pentosaceus SNSS18

**Figure 7:**
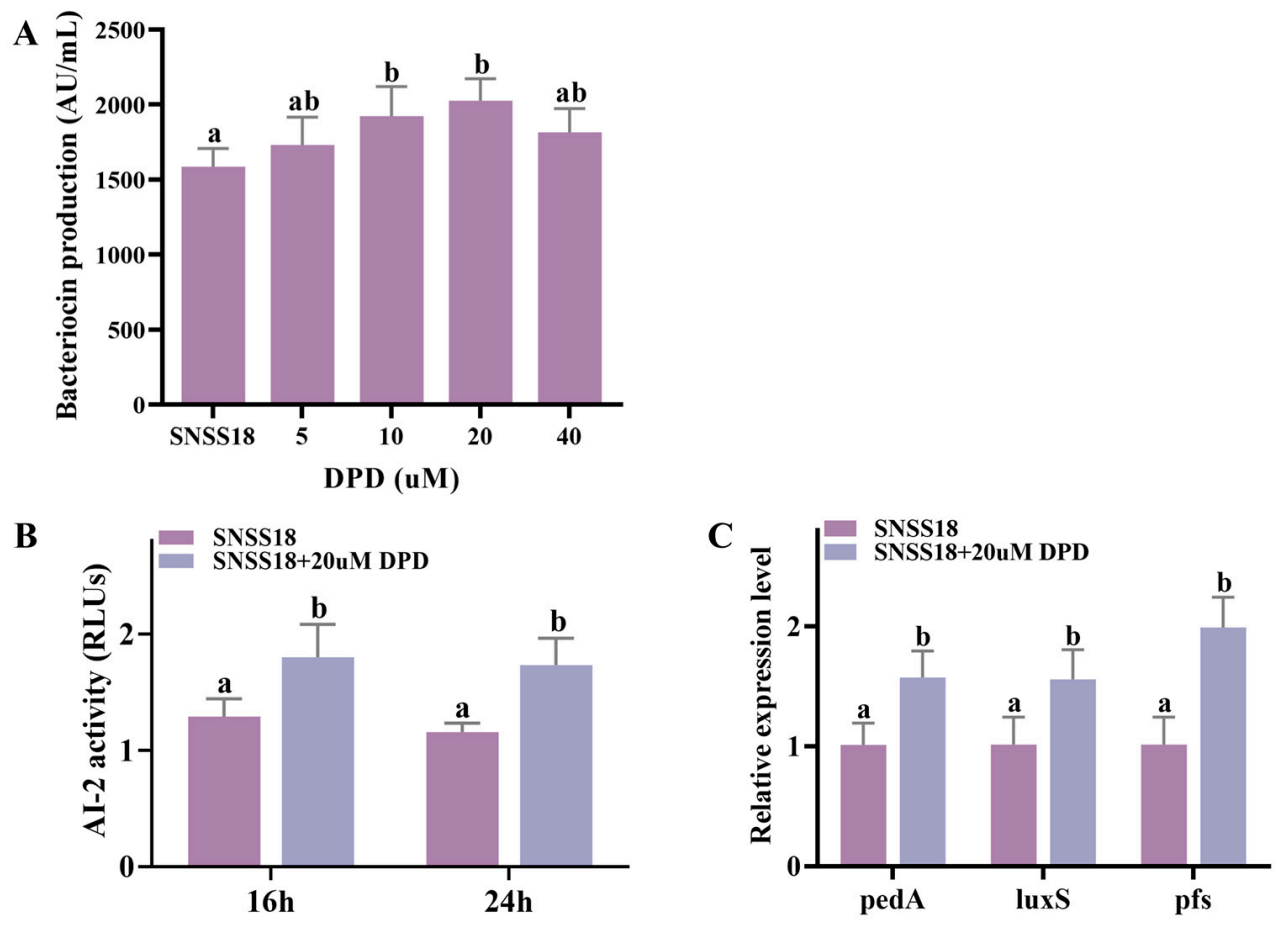
Effects of exogenous DPD on bacteriocin production (**A**), AI-2 activity (**B**), and the expression of *pedA*, *luxS*, and *pfs* (**C**) in *P. pentosaceus* SNSS18. Different letters indicate significant differences between groups (*p* < 0.05).

As shown in Figure 7A, DPD (the precursor of AI-2) supplementation affected pediocin production in a concentration-dependent manner. DPD at 10 and 20 µM significantly increased pediocin production, with 20 µM showing the strongest effect, whereas a higher concentration (40 µM) resulted in reduced induction. This concentration-dependent response suggests that an optimal AI-2 level may be required for maximal activation of the LuxS/AI-2 QS pathway, while excessive signal accumulation may lead to signal saturation or feedback regulation. Consequently, AI-2 activity and the expression of related genes were further examined. AI-2 activity and the expression of *pedA*, *luxS*, and *pfs* were significantly increased upon addition of 20 µM DPD (Figure 7B,C). These results indicated that exogenous DPD and AI-2 function similarly to aspartate, cysteine, methionine, and uracil in regulating the behavior of *P. pentosaceus* SNSS18. Thus, these amino acids and nucleobases might modulate pediocin production, at least in part, via the LuxS/AI-2 QS system.

However, amino acids and nucleobases are essential metabolites that integrate carbon, nitrogen, and energy metabolism in LAB, playing complex roles. Beyond their potential role in the LuxS/AI-2 QS system, they can also affect the production of secondary metabolites by serving as biosynthetic substrates, providing energy, and modulating key enzymatic activities [19,23,46]. Thus, the precise regulatory mechanisms through which aspartate, cysteine, methionine, and uracil enhance pediocin production in *P. pentosaceus* SNSS18—including the interplay between the LuxS/AI-2 system and other metabolic pathways—require further elucidation.

## 4. Conclusions

This study identified the gene clusters for pediocin PA-1 biosynthesis and the LuxS/AI-2 QS system in *P. pentosaceus* SNSS18. Supplementation with methionine (0.5 g/L) or cysteine (0.5 g/L) enhanced AI-2 synthesis and upregulated the LuxS/AI-2 QS system, while aspartate (1 g/L) and uracil (1.5 g/L) exhibited similar but weaker effects, contributing to the significant increase in pediocin production by *P. pentosaceus* SNSS18 at least in part. Conversely, alanine (0.5 g/L) and adenine (1 g/L) appeared to enhance pediocin production through LuxS/AI-2 QS-independent mechanisms. These effects may involve alanine-derived metabolic shifts that influence global transcriptional regulators such as CodY and CcpA, as well as stress responses triggered by adenine-induced growth inhibition. However, the regulatory pathways involving these amino acids and nucleobases are complex, and further investigation is required to elucidate their integrated regulatory networks, including QS-dependent and alternative signaling pathways. In summary, this work demonstrates that exogenous supplementation with specific amino acids and nucleobases is an effective strategy to enhance bacteriocin yield, and the pediocin biosynthesis in *P. pentosaceus* SNSS18 is regulated, at least in part, by the LuxS/AI-2 QS system.

## Figures and Tables

**Table 1:** Primer sequences for RT-qPCR analysis.

Genes		Oligonucleotide Sequence (5′-3′)
*16S rRNA*	Forward	CGTAGGTGGCAAGCGTTATCC
	Reverse	GCACTCAAGTCTCCCAGTTTCC
*pedA*	Forward	TGTGGCAAACATTCCTGCTC
	Reverse	TGATGTCCACCAGTAGCCCA
*luxS*	Forward	CTGGATTATTGCGAGATCGAATGG
	Reverse	ACTTCTGTGGTTGTAGGTTCTCC
*pfs*	Forward	GTTCGGGAATCGGTAAAGTTGAG
	Reverse	ACGCCACTTCAGTTGATAGCA

**Table 2:** Effects of different concentrations of amino acids and nucleobases on the bacteriocin production by *P. pentosaceus* SNSS18.

Exogenous Substance	Concentration (g/L)	Bacteriocin Production (AU/mL)
SNSS18	0	1591.1 ± 91.13
SNSS18 + Ala	0.1	1962.06 ± 91.96 *
	0.5	2200.00 ± 147.87 **
	1	1789.80 ± 156.61
SNSS18 + Asp	0.5	1901.77 ± 136.84 *
	1	2258.16 ± 193.03 **
	1.5	1679.95 ± 125.11
SNSS18 + Cys	0.1	2179.10 ± 66.07 **
	0.5	2380.19 ± 125.45 **
	1	1534.20 ± 63.95
SNSS18 + Met	0.1	2031.29 ± 75.05 *
	0.5	2080.17 ± 197.37 *
	1	1925.44 ± 176.58 *
SNSS18 + Ura	0.5	2142.12 ± 180.43 **
	1	2219.35 ± 128.19 **
	1.5	2557.68 ± 167.20 **
	2	2287.94 ± 107.64 **
SNSS18 + Ade	0.5	2038.77 ± 104.46 *
	1	2364.92 ± 165.34 **
	1.5	1874.26 ± 74.29 *

* *p* < 0.05, ** *p* < 0.01 compared with the control (SNSS18).

## Data Availability

The original contributions presented in the study are included in the article/Supplementary Materials; further inquiries can be directed to the corresponding author.

## Supplementary Materials

The following supporting information can be downloaded at https://www.mdpi.com/article/10.3390/foods15152739/s1, Table S1: Overview of gene functional annotations of *P. pentosaceus* SNSS18; Figure S1: Effects of different amino acids and nucleobases on the bacteriocin production by *P. pentosaceus* SNSS18; Figure S2: Phylogenetic tree based on 16S rRNA gene (A) and housekeeping genes (B); Figure S3: Linear map of the fusaricidin B biosynthetic gene cluster.
