# Supplementary material for: Enhancement of Pediocin Production in Pediococcus pentosaceus SNSS18 by Exogenous Amino Acids and Nucleobases: A Mechanistic Elucidation
Source: Foods. 2026 Aug 4;15(15):2739. doi: 10.3390/foods15152739 (PMC13465018; doi:10.3390/foods15152739)
Supplement: Supplementary file 1 [file foods-15-02739-s001.zip › foods-4431009-supplementary.pdf]

Table S1. Overview of gene functional annotations of *P. pentosaceus* SNSS18.

Database	NR	Pfam	COG	GO	KEGG
Gene Number	1801	1545	1481	1072	1337

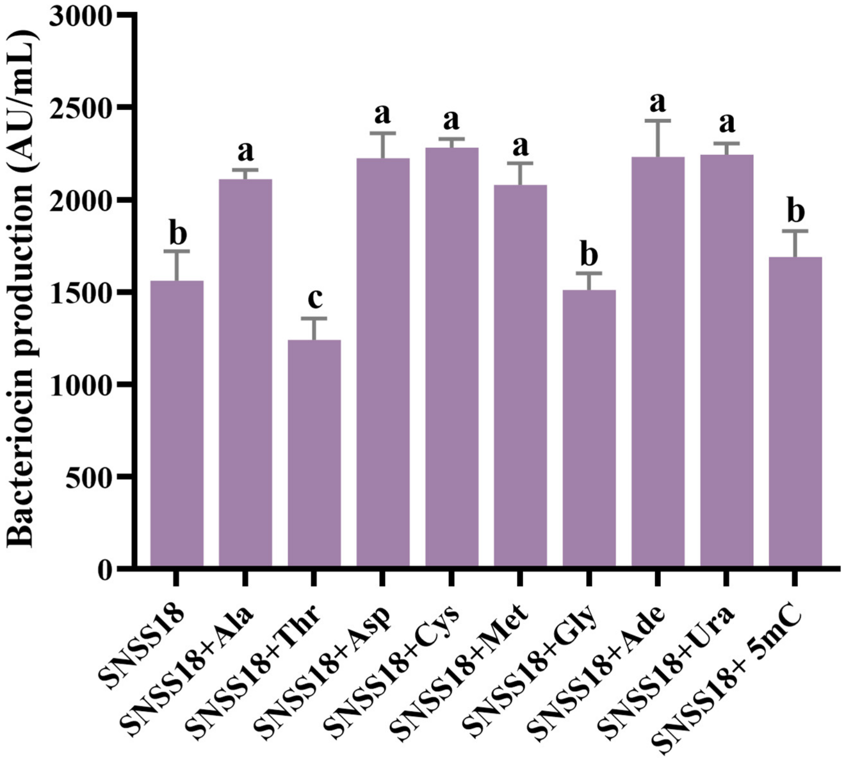

Figure S1. Effects of different amino acids and nucleobases on the bacteriocin production by *P. pentosaceus* SNSS18.

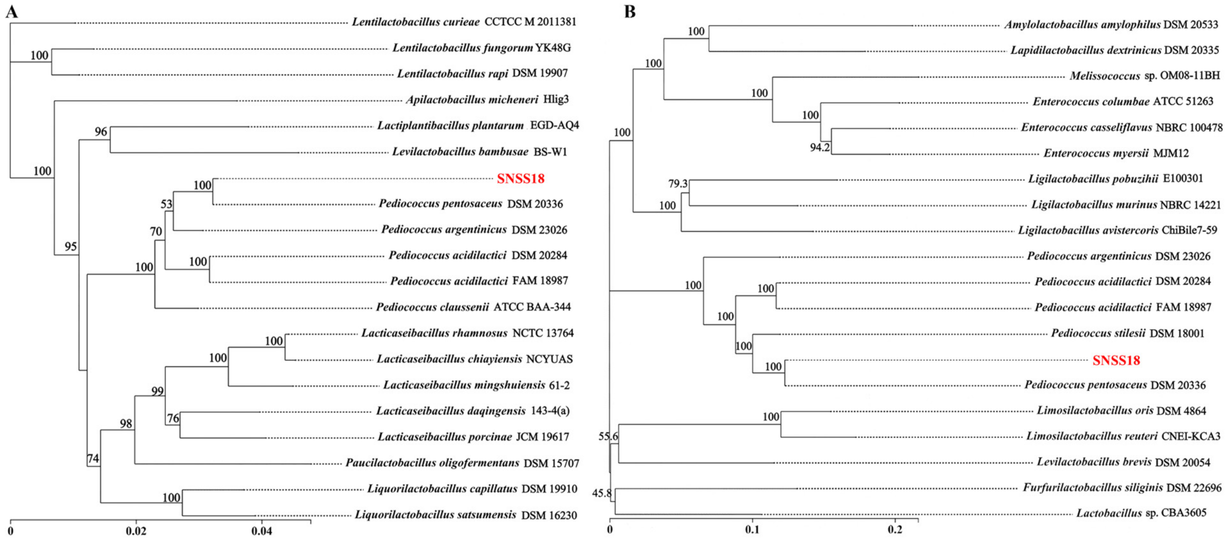

Figure S2. Phylogenetic tree based on 16S rRNA gene (A) and housekeeping genes (B).

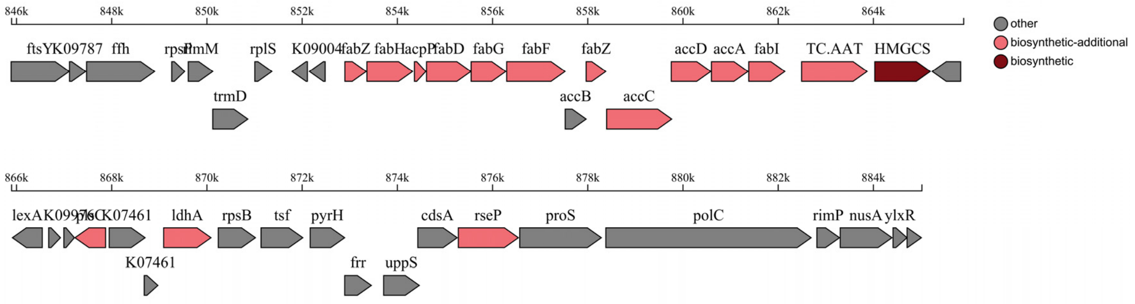

Figure S3. Linear map of the fusaricidin B biosynthetic gene cluster.
